# Hovering in the heat: effects of environmental temperature on heat regulation in foraging hummingbirds

**DOI:** 10.1098/rsos.171056

**Published:** 2017-12-06

**Authors:** Donald R. Powers, Kathleen M. Langland, Susan M. Wethington, Sean D. Powers, Catherine H. Graham, Bret W. Tobalske

**Affiliations:** 1 Department of Biology, George Fox University, Newberg, OR, USA; 2 Hummingbird Monitoring Network, Patagonia, AZ, USA; 3 Swiss Federal Research Institute (WSL), 8903 Birmensdorf, Switzerland; 4 Field Research Station at Fort Missoula, Division of Biological Sciences, University of Montana, Missoula, MT, USA

**Keywords:** heat dissipation, hovering, hummingbirds, infrared thermography, body-heat regulation, thermoregulation

## Abstract

At high temperature (greater than 40°C) endotherms experience reduced passive heat dissipation (radiation, conduction and convection) and increased reliance on evaporative heat loss. High temperatures challenge flying birds due to heat produced by wing muscles. Hummingbirds depend on flight for foraging, yet inhabit hot regions. We used infrared thermography to explore how lower passive heat dissipation during flight impacts body-heat management in broad-billed (*Cynanthus latirostris*, 3.0 g), black-chinned (*Archilochus alexandri*, 3.0 g), Rivoli's (*Eugenes fulgens*, 7.5 g) and blue-throated (*Lampornis clemenciae*, 8.0 g) hummingbirds in southeastern Arizona and calliope hummingbirds (*Selasphorus calliope*, 2.6 g) in Montana. Thermal gradients driving passive heat dissipation through eye, shoulder and feet dissipation areas are eliminated between 36 and 40°C. Thermal gradients persisted at higher temperatures in smaller species, possibly allowing them to inhabit warmer sites. All species experienced extended daytime periods lacking thermal gradients. Broad-billed hummingbirds lacking thermal gradients regulated the mean total-body surface temperature at approximately 38°C, suggesting behavioural thermoregulation. Blue-throated hummingbirds were inactive when lacking passive heat dissipation and hence might have the lowest temperature tolerance of the four species. Use of thermal refugia permitted hummingbirds to tolerate higher temperatures, but climate change could eliminate refugia, forcing distributional shifts in hummingbird populations.

## 1. Introduction

Increases in daily and yearly temperature variation associated with climate change might make body-temperature maintenance increasingly difficult for birds. Low mechanical efficiency of flight muscles results in substantial heat generation during flight in birds [1,2] that must be dissipated to avoid hyperthermia [3,4]. Dissipating excess heat produced during flight is challenging for birds because feathers provide an insulative layer that restricts heat loss, particularly at slow flight speeds when forced convection is low [3–6]. The degree to which plumage restricts evaporative heat dissipation during flight is unknown, but will probably not be a substantial barrier [7]. However, we know that birds passively dissipate heat (radiation, conduction and convection) through specific heat dissipation areas (HDAs) around the eyes, shoulder and feet/legs where plumage density is low, thereby exposing the skin [3,4,6]. In calliope hummingbirds (*Selasphorus calliope*), these HDAs allow sufficient passive heat dissipation for maintenance of heat balance at a moderate environmental temperature (21°C) across flight speeds in the range 0–12 m s^−1^ [3]. At higher environmental temperatures, the thermal gradient that drives passive heat dissipation will become smaller or be eliminated, making heat dissipation difficult.

When environmental temperature becomes sufficiently high to eliminate passive heat dissipation, the only avenue for heat loss in flying birds is evaporative water loss (EWL). The ability to use EWL to compensate for the loss of passive heat dissipation will probably vary among bird species based on their physiological and ecological characteristics [7–9]. In resting birds, rates of EWL vary as a function of skin exposure, lipid deposition in the skin, panting, morphological structures such as waddles and combs, and vascular enhancement of the buccal cavity [7,10,11]. However, the functional significance of such controls has not been studied in flight. Measurements of EWL in flying birds are few, and most that do exist were made at relatively low ambient temperature and on birds greater than 75 g [12–15]. Smaller species, including hummingbirds, are sensitive to environmental temperature [16–18] and therefore at high temperature must have the ability to increase EWL during flight if they are to engage in flight.

Birds must cope with a range of daily and annual temperatures. In warm environments, some hummingbirds avoid high temperatures by reducing their activity during the middle of the day (S. Wethington 2013, unpublished point count data). In thermally complex habitats, birds can adjust behaviour to take advantage of cooler refugia to slow the impact of increasing environmental temperature [19]. However, if environmental temperatures within even extreme refugia exceed the point where passive heat dissipation is possible, then birds must rely on evaporative heat dissipation during flight activity for continued existence in their habitat. Therefore, studies of heat dissipation during hovering flight are important if we are to anticipate when thermoregulation will make it challenging for hummingbirds to acquire energy and fulfil their important ecological role as pollinators.

Hummingbirds are an excellent model for the study of heat dissipation during flight because they can live in hot climates [20,21], and typically hover while foraging. The power required for hovering is extremely high [3,22], resulting in substantial heat generation. Because hummingbirds are stationary during hovering, they do not benefit from convective heat loss except for that resulting from air movement generated by their wings [3]. The magnitude of this flow near HDAs is less than 2.5 m s^−1^ [3], whereas hummingbirds are capable of forward flight speeds of greater than 12 m s^−1^ [23,24]. At high environmental temperatures, when there is no thermal gradient to support passive heat dissipation, the ability to hover, and thus forage, would be dependent on evaporative heat dissipation. However, it is not clear at what temperature hummingbirds lose the ability to compensate for the loss of passive heat dissipation using EWL. Respiratory evaporative water loss (REWL) in hummingbirds accounts for less than 40% of total heat dissipated during hovering [25]. Thus, cutaneous evaporative water loss (CEWL) would need to account for the balance if hummingbirds are to maintain a balanced heat budget. While plumage is a barrier to passive heat dissipation during flight, it might not restrict EWL. CEWL in flying birds at high temperature has not been studied, but plumage does not appear to be a barrier in resting columbids even when temperature is greater than 50°C [7]. If there is no restriction on CEWL during hovering, then evaporative heat dissipation could allow hummingbirds to be active at high temperatures.

In this study, we use measurements of operative temperature [26] and infrared (IR) thermography on captive and free-living hummingbirds to examine how the thermal gradient associated with passive heat dissipation from the HDAs in hummingbirds changes over a range of environmental temperatures. We address three questions. (i) At what environmental temperature is the thermal gradient for heat dissipation eliminated? As the thermal gradient for passive heat dissipation is eliminated when environmental temperature and the temperature of the dissipating surface are equal, we hypothesize that passive heat dissipation will be eliminated when the environmental temperature equals the surface temperature of the HDAs (approx. 36°C) [3]. (ii) Do hummingbirds that currently live in warm southeastern Arizona habitats during summer experience extended periods where they must rely on evaporative heat dissipation during hovering due to loss of the thermal gradient? Given midday summer temperatures, we hypothesize that our study species will be constrained for several hours during the day. (iii) Do hummingbirds exhibit physiological adaptation and/or behaviour adjustment when passive heat dissipation is eliminated, thereby tolerating periods of high daytime temperature? Because hummingbirds must feed regularly to meet their energy demand, we hypothesize that species will exhibit higher than expected temperature tolerance (physiological adjustments) and/or spend increased time in cooler thermal refugia (behavioural) during the hottest parts of the day.

## 2. Methods

### 2.1. Study sites

Field measurements were conducted at four sites in southeastern Arizona May–August 2013 and 2014. Two study sites were near the town of Patagonia, Santa Cruz County. Harshaw Creek (HC; N31 29 56.0, W110 40 55.6), with an elevation range of 1375–1635 m, had riparian vegetation along HC that included large sycamores (*Platanus wrightii*) surrounded by oak–mesquite (*Quercus* sp., *Prosopis* sp.) at higher elevations. Sonoita Creek State Natural Area (SC; N31 29 42.8, W110 51 24.1), elevation 1150–1250 m, was characterized by riparian vegetation along Sonoita Creek that included large sycamores, cottonwood, ash and willow surrounded by high desert vegetation including mesquite, acacia, ocotillo, yucca and various cactus species. HC is climatically influenced by the Sonoran Desert, while SC is a transition zone between the Sonoran and Chihuahuan Deserts. At both sites the environmental temperature approaches 50°C during summer.

Two higher-elevation sites were located in the Chiricahua Mountains, Cochise County. El Coronado Ranch (EC; N31 52 12.9, W109 21 48.9) is on the western slope of the Chiricahua Mountains and at an elevation range of 1700–2050 m. The site consisted of a variety of habitats that included various mixtures of pine, juniper, oak, a few other broad-leaf species and a small amount of grassland. The Southwestern Research Station (SWRS; N31 52 58.7, W109 12 19.2) is on the eastern slope of the Chiricahua Mountains at an elevation range of 1640–1900 m. The site included sycamores, alder and willows in the riparian zones surrounded by pine–oak or pine–juniper forest [27].

IR thermography measurements of the mean surface temperature (*T*_body_) and the mean HDA surface temperature (*T*_HDA_) on captive birds were made at HC and SWRS in southeastern Arizona and at the University of Montana Flight Lab, Missoula, Montana (Missoula County, N46 50 22.4 W114 03 09.0, elevation 961 m). Temperature-related symbols used in this study are defined in table 1.

**Table 1.** Definitions for symbols used in this paper.

symbol	definition
*T*_a_	ambient temperature
*T*_body_	mean whole-body surface temperature
*T*_e_	operative temperature
*T*_eye_	mean surface temperature of the eye heat dissipation area
*T*_HDA_	mean surface temperature across all heat dissipation areas
Δ*T*	thermal gradient for passive heat dissipation
Δ*T*_0_	the temperature at which Δ*T* = 0

### 2.2. Study species

At HC and SC, we studied captive and free-living broad-billed hummingbird (*Cynanthus latirostris*, 3.3 g; BBLH) and black-chinned hummingbird (*Archilochus alexandri*, 3.0 g; BCHU) June–July 2013. At both EC and SWRS, we studied Rivoli's hummingbirds (*Eugenes fulgens*, 7.5 g; RIHU) and BCHU, and at SWRS blue-throated hummingbirds (*Lampornis clemenciae*, 8.0 g; BLUH). Laboratory studies on captive birds were done at SWRS during June 2013, and studies of free-living birds done at both EC and SWRS during June–July 2014. All Arizona species arrive on site from Mexico in spring and are present through most of summer [20,28–30]. Calliope hummingbirds (*Selasphorus calliope*; three males, 2.4–2.9 g; four females, 2.6–3.1 g; CAHU) were studied in the laboratory during June 2010–2012 in Missoula, MT (Missoula County), USA.

For our laboratory experiments, birds were housed individually in 1 m × 1 m × 1 m cages and fed a 50 : 50 mixture of 20% sucrose solution and Nectar Plus© ad libitum. All study subjects maintained mass during captivity, confirming that the feeder solution adequately supplied essential dietary needs. All protocols associated with hummingbird care and experimentation were approved by the George Fox University IACUC (P107) and the University of Montana IACUC (002–12BTDBS-012012). Collection of hummingbirds for our laboratory and field studies was authorized by the US Fish and Wildlife Service (Arizona permit no. MB75714A-0, Montana permit no. MB771277-0), Arizona Department of Game and Fish (permit nos. SP609587, SP674472) and Montana Fish, Wildlife, and Parks (permit no. 2012-34).

### 2.3. Operative temperature

We measured operative temperature (*T*_e_) using Cu–Cn thermocouples (type-T) inside hollow copper spheres (0.53 cm^3^) painted flat grey [31] and placed throughout typical hummingbird habitat across vegetation and elevation gradients. Each sphere was attached to a wooden stake 1 m above the ground. Spheres were connected to either Campbell 21× or Veriteq Spectrum 1700 temperature loggers set to record *T*_e_ at 15 min intervals. For analysis of IR thermography data (see below) we used the site-specific mean *T*_e_ recorded closest to the time data were collected (within ± 7.5 min).

### 2.4. Infrared thermography

To measure surface temperature of hovering hummingbirds, we recorded IR images using a FLIR SC6700 IR video camera (640 × 480 pixel resolution, sampling at 300 Hz). Recordings were made of both free-living hummingbirds at a feeder and captive birds housed in a 1 m^3^ Plexiglas® enclosure that featured an open-mesh floor. Calibration images of a 12 cm ruler were recorded prior to each flight to set the scale used for measurements of single-frame images. Videos of captive birds were recorded through a hole cut in the Plexiglas® wall of the enclosure. Captive hummingbirds were positioned for recording in the enclosure using a 10.0 ml feeder made from a Luer-lock syringe containing 20% sucrose solution. Free-living birds were recorded hovering in front of a feeder prior to feeding. The IR camera was placed 1 m from outdoor feeders with the camera lens level with the feeder base. Feeders were placed in complete shade to avoid measurement errors due to sunlight reflection. All recordings were a lateral view, with wings at near end upstroke visualizing half the hummingbird's surface. We assumed that emissivity was 0.95 across all surfaces of the hummingbirds [32]. For each recording we exported single-frame images illustrating end upstroke for analysis. Videos were recorded using the ExaminIR or ResearchIR software (FLIR, Inc.), and single-frame images were analysed using ImageJ (NIH).

For captive birds, each single-frame image was analysed for whole-body (plumage) surface temperature (*T*_body_,°C), HDA surface temperature (T_HDA_,°C) and ambient temperature (*T*_a_,°C); *T*_a_ was measured by recording an IR image of a black cloth that lined the rear wall of the chamber. This method of measuring *T*_a_ was validated with concurrent measurements of internal chamber *T*_a_ using a type-T (Cu–Cn) thermocouple; *T*_body_ was measured by tracing the outline of the lateral body view in each IR image; and *T*_HDA_ was an integrated measure of the traced eye, axial and feet/legs HDAs (see Powers *et al.* [3] for a description of HDAs). For free-living birds, we measured *T*_body_, and estimated the eye HDA surface temperature (*T*_eye_) and length along a transect from the base of the bill, through the eye, to the back of the head. We used a transect because at high temperature the boundary of the eye HDA becomes obscured. Boundaries for the eye HDA were assumed to be where the surface temperature along the transect increased above *T*_body_ (excluding the eye). For each of the temperature measurements described above, mean, minimum and maximum values were recorded.

### 2.5. Thermal gradient

The thermal gradient for passive heat transfer (Δ*T*) was estimated as Δ*T* = *T*_HDA_ − *T*_e_ in captive birds, and Δ*T* = *T*_eye_ − *T*_e_ in free-living birds. The Δ*T* values calculated when *T*_eye_ < *T*_e_ are shown graphically, but not included in regression models because *T*_eye_ cannot be accurately measured under these conditions.

### 2.6. Analysis

We analysed the relationship between *T*_body_/*T*_HDA_ and *T*_a_ measured in captive hummingbirds using phylogenetic general linear mixed models (Hierarchial Bayesian MCMCglmm, MCMCglmm R package [33]). The hummingbird phylogeny generated by McGuire *et al*. [34] was used to estimate the phylogenetic structure. Within species, we used linear least-squares regression to model the relationship between *T*_body_/T_HDA_ and *T*_a_, Δ*T*/length of eye HDA and *T*_e_, and Δ*T* and length of the eye HDA. Because the relationship between Δ*T* and the length of the eye HDA was nonlinear, we log-transformed the data. We tested for interspecific difference between slopes of linear regressions using ANCOVA.

## 3. Results

**Figure 1.**
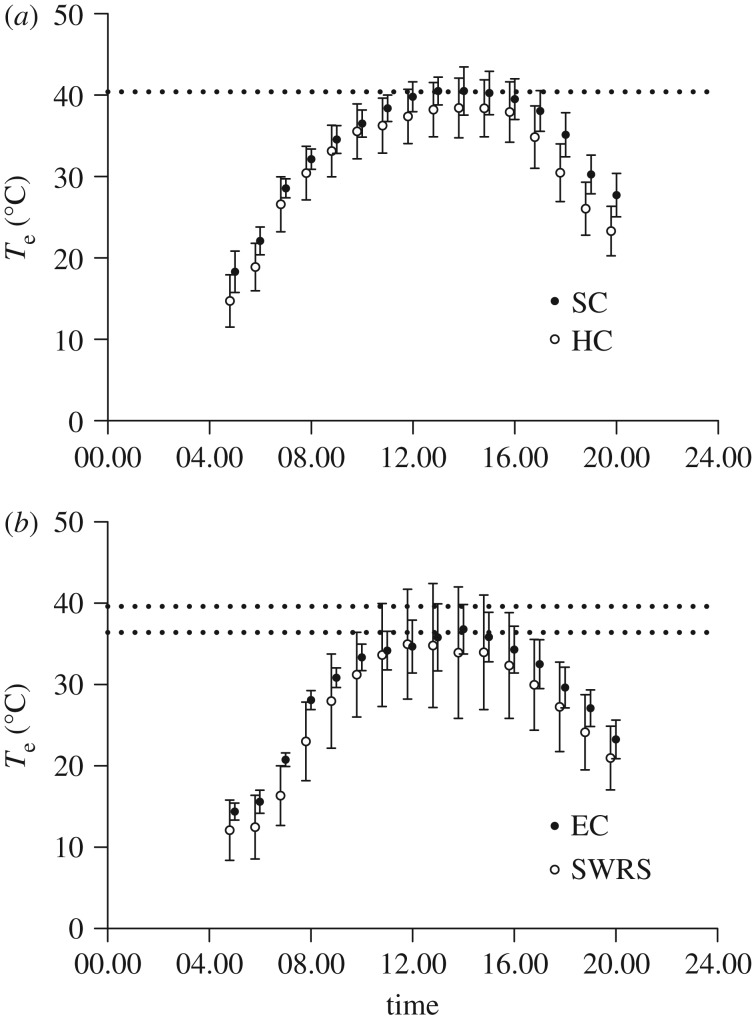
Daytime hourly *T*_e_ (mean ± s.d.) for each study site. For the Patagonia sites (*a*), the dotted line is where *T*_e_ = Δ*T*_0_ for broad-billed hummingbirds. For the Chiricahua sties (*b*), the two dotted lines represent the range of Δ*T*_0_ for species studied at these sites. The upper dotted line is Δ*T*_0_ for black-chinned hummingbirds and the lower dotted line Δ*T*_0_ for blue-throated hummingbirds.

Minimum and maximum daytime *T*_e_ at both Patagonia and the Chiricahuas differed by greater than 20°C, with maximums at SC in Patagonia occasionally near 50°C. In Patagonia, at HC the mean hourly temperature was cooler and more variable than SC. In the Chiricahuas, the mean hourly temperature was cooler at SWRS than EC except during 11.00–12.00. Hourly temperature was also more variable at SWRS (figure 1).

**Figure 2.**
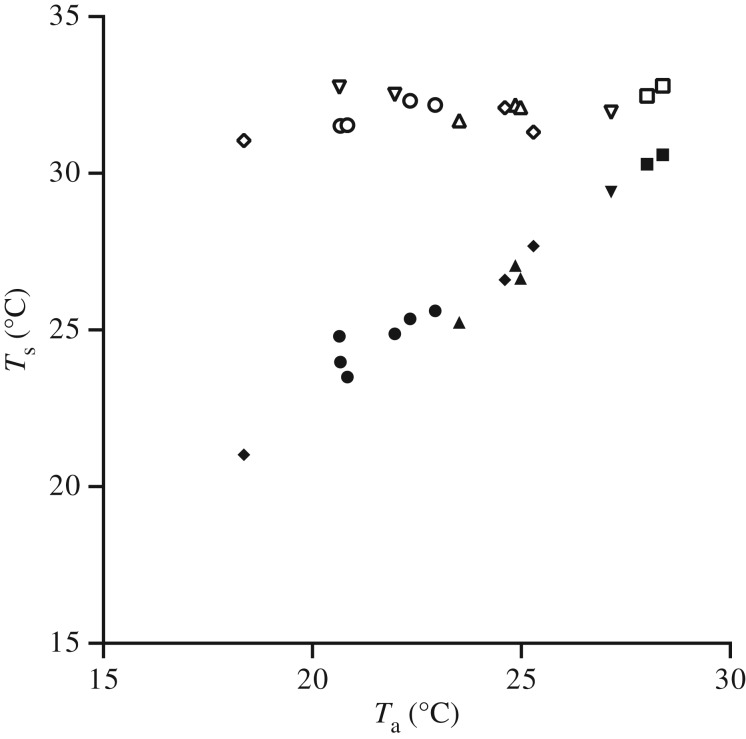
Mean *T*_body_ (closed symbols) and *T*_HDA_ (open symbols) as a function of *T*_a_ for captive hummingbirds ranging from 2 to 8 g. MCMCglmm models show that mean *T*_body_ was positively correlated with *T*_a_, while mean *T*_HDA_ did not change with *T*_a_, and was probably controlled by endogenous heat production. Symbols are calliope hummingbird (inverted triangle), black-chinned hummingbird (square), broad-billed hummingbird (circle), magnificent hummingbird (triangle) and blue-throated hummingbird (diamond).

Bayesian MCMCglmm, including phylogeny to estimate statistical significance, showed that laboratory measurements of *T*_body_ during hovering in captive birds was significant and positively correlated with *T*_a_ in the range of 18–30°C (figure 2; electronic supplementary material, table S1, *p*_MCMC_ < 0.001). Over the same *T*_a_ range the integrated mean *T*_HDA_ showed no relationship with *T*_a_ (figure 2; electronic supplementary material, table S1, *p*_MCMC_ < 0.08). Assuming a mean model intercept for all species included in the analysis, the size of the thermal gradient (Δ*T*) was negatively correlated with *T*_a_ (figure 2; *R*^2^ = 0.971), and ranged from 12.7°C at *T*_a_ = 18.4°C to 4.4°C at *T*_a_ = 28.4°C (Δ*T* = 30.21–0.92*T*_a_).

**Figure 3.**
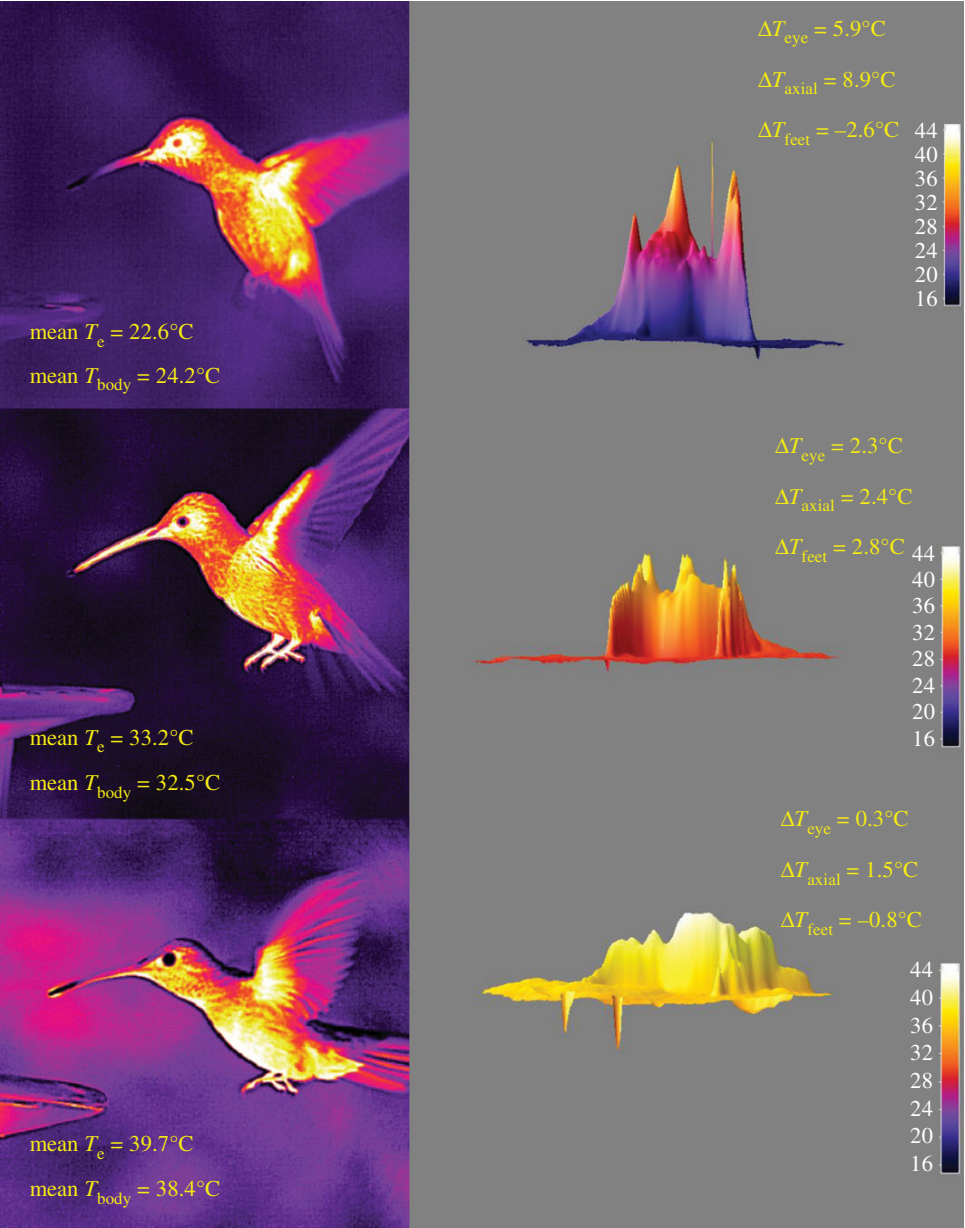
Lateral-view infrared (IR) images of a hovering broad-billed hummingbird at *T*_e_ in the range of 22.6–39.7°C and corresponding values of *T*_body_. On the right are three-dimensional surface plots of *T*_body_ constructed from the adjacent IR images along with estimated Δ*T* for eye, axial and feet HDAs.

IR images of free-living hummingbirds show that the relationships between *T*_e_ and both *T*_body_ and *T*_eye_ are consistent with laboratory observations. Typical lateral IR images and corresponding three-dimensional surface plots for BBLH are shown in figure 3. Three-dimensional surface plots, as well as estimations of Δ*T* confirm that Δ*T* was negatively correlated with *T*_e_, and that when *T*_e_ approaches 40°C, there was no longer a sufficient Δ*T* to support passive heat dissipation.

**Figure 4.**
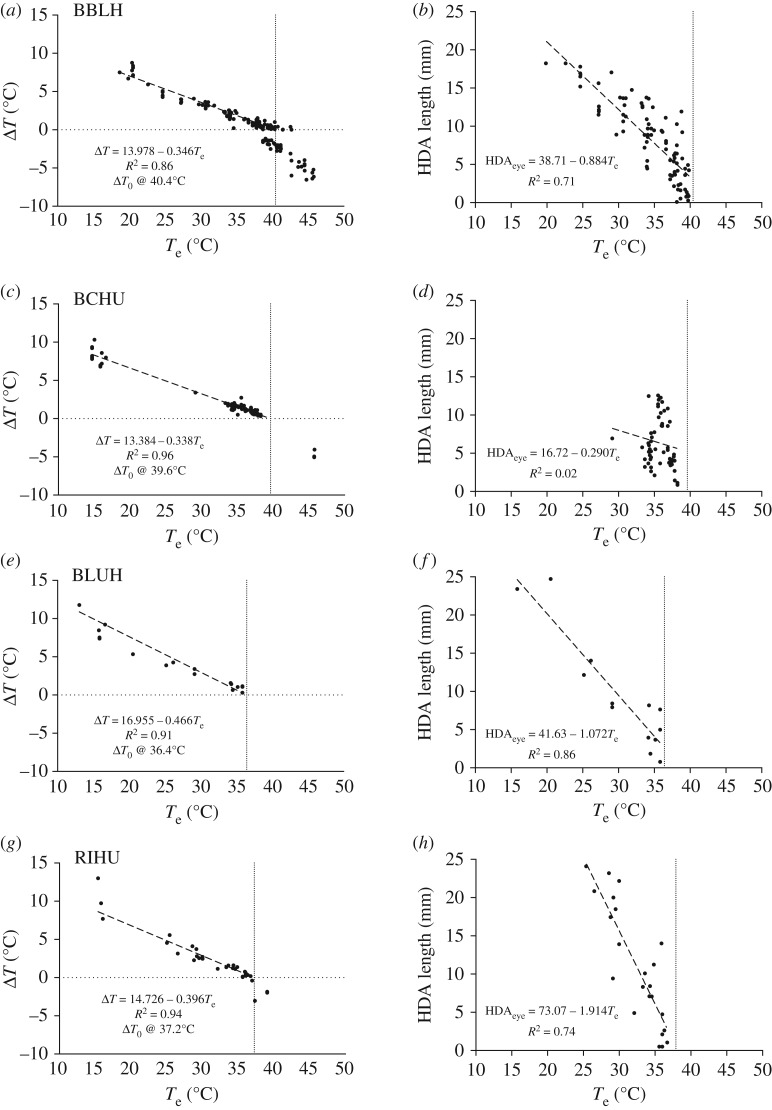
(*a*,*c*,*e*,*g*) Estimated Δ*T* as a function of *T*_e_. Dashed lines are the linear least-squares regression models for Δ*T* values >0, and values for Δ*T* < 0 are shown, but their accuracy was less certain due to the difficulty of separating the eye HDA from general plumage surfaces. Vertical dotted lines indicate Δ*T*_0_. All regressions for Δ*T* versus *T*_e_ are statistically significant (*p* < 0.05), and slopes differ significantly among species (*F*_3,210_ = 11.45, *p* < 0.001). (*b*,*d*,*f*,*h*) Summed length of sections of the transect through the eye HDA, where *T*_eye_ > *T*_e_ plotted as a function of *T*_e_. Regressions are all statistically significant (*p* < 0.05) except for black-chinned hummingbirds (*p* = 0.27), and slopes differ significantly among species (*F*_4,260_ = 51.89, *p* < 0.0001).

Body size had a significant effect upon Δ*T* and *T*_e_ among species (figure 4). For BBLH and BCHU (smaller species), regression models predict that Δ*T* = 0 (Δ*T*_0_) when *T*_e_ is 40.4°C and 39.6°C, respectively (figure 4). For BLUH and RIHU (larger species), regression models predict Δ*T*_0_ at 36.4°C and 37.2°C, respectively. The slope of the regression models was similar for BBLH and BCHU, and was lower than the slopes for the larger species. Similar slopes and Δ*T*_0_ values suggest that BBLH and BCHU respond similarly to increasing temperatures. The slope for BLUH was 27% greater than that of the smaller species, and 15% greater than that for RIHU. BLUH also had the lowest Δ*T*_0_, suggesting elimination of passive heat dissipation at the lowest *T*_e_ observed in our study species. Regression models for eye HDA length when Δ*T *> 0 were statistically significant for all species except BCHU. The regression model for RIHU had a notably higher slope than either BBLH or BLUH, suggesting rapid reduction in the size of the eye HDA.

**Figure 5.**
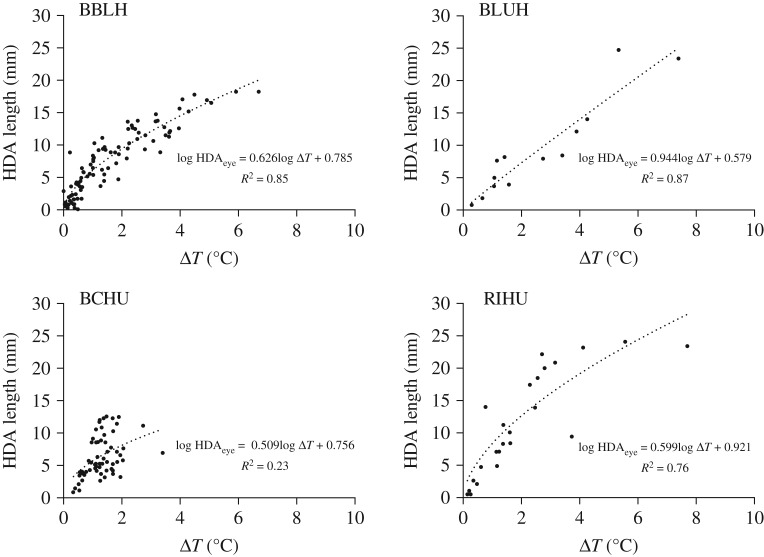
The relationship between the summed portion of the eye HDA transect, where *T*_eye_ > *T*_e_ as a function of Δ*T*. Regressions (dotted lines) are fitted to non-log-transformed data to show that both eye HDA size and decline in Δ*T* contribute to the rapid elimination of passive heat dissipation as *T*_e_ nears Δ*T*_0_. Log-transformed regression models differ among species (*F*_6,183_ = 7.71, *p* < 0.0001).

Eye HDA length and Δ*T* were positively correlated in all species (figure 5), but in BBLH, BCHU and RIHU the relationship was nonlinear, so regression models were run on log–log-transformed data. Nonlinearity was probably due to transition along the transect from high-plumage surface (environmentally controlled *T*_body_) into the eye HDA proper (physiologically controlled *T*_HDA_).

**Figure 6.**
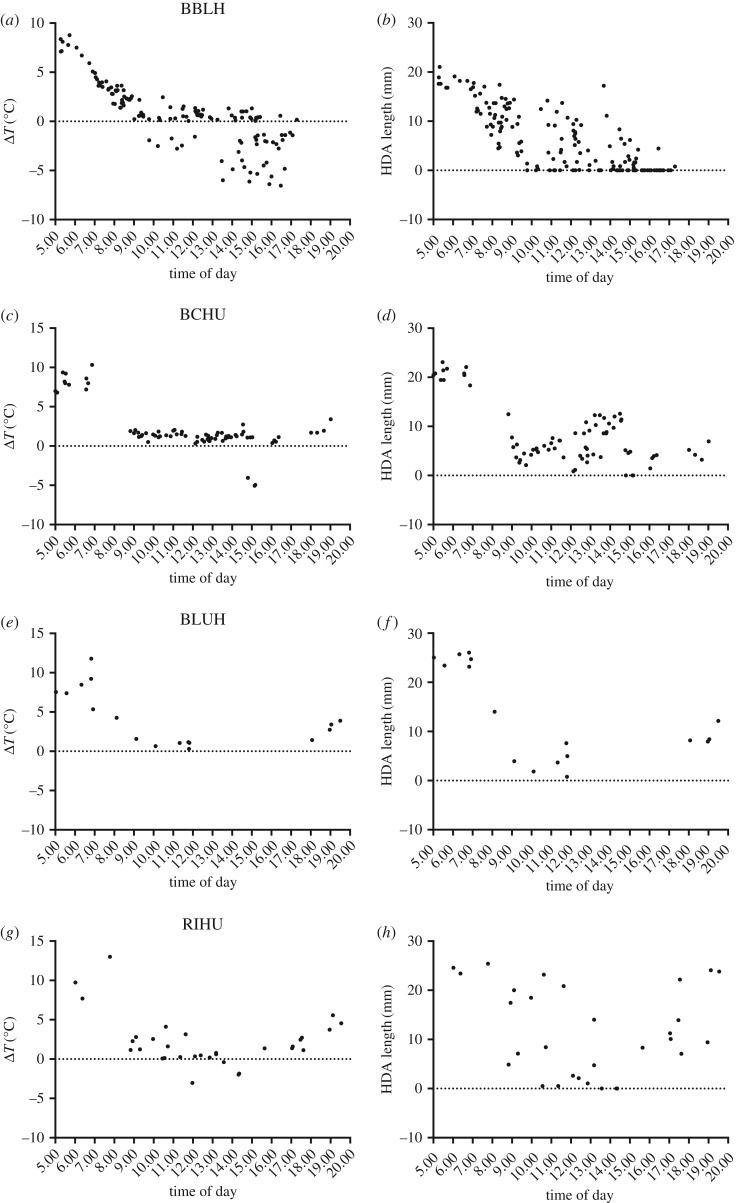
Changes in Δ*T* (*a,c,e,g*) and eye HDA length (*b,d,f,h*) during daylight hours. Dotted lines represent Δ*T*_0_ and eye HDA length equal to 0. Negative values for Δ*T* indicate reversal of the thermal gradient and likely periods of passive heat gain.

There was variation in daytime Δ*T* and eye HDA length (figure 6). For BBLH, Δ*T* was often unfavourable for passive heat dissipation during 09.00–17.00, although high temperature was more routine at SC than HC (figure 6*a*). The length of the eye HDA was variable probably due to the rapid rate of decrease in eye HDA length when Δ*T* is small (figure 5). Variable eye HDA length for much of the day was characteristic for all species. BCHU benefitted from a higher Δ*T*_0_, and were consistently able to maintain a small, but favourable Δ*T* throughout the day (figure 6*b*). Although we had relatively few measurements of BLUH (*n* = 17), it was interesting that data were absent from 12.00 to 18.00, which includes periods of the day when Δ*T* was unfavourable. Unlike BLUH, RIHU were more active midday (12.00–15.00) even though *T*_e_ was near Δ*T*_0_ (figure 6*d*).

**Figure 7.**
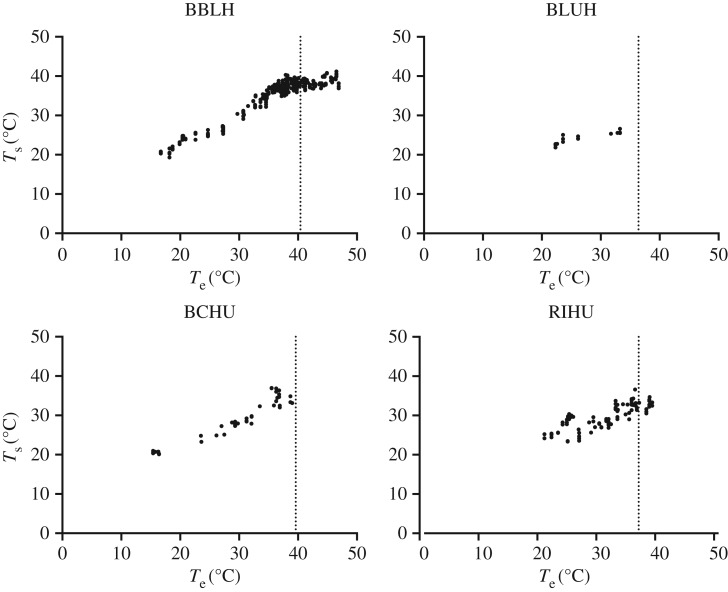
*T*_body_ as a function of *T*_e_ in free-living hummingbirds over the range of temperatures measured in this study. Vertical dotted lines indicate the position of Δ*T*_0_ for each species.

For free-living birds *T*_body_ increased with *T*_e_ until *T*_e_ was near Δ*T*_0_ (figure 7). In BBLH, the linear regression slope of *T*_body_ versus *T*_e_ when *T*_e_ < Δ*T*_0_ was 0.824 and was significantly shallower at 0.156 when *T*_e_ > Δ*T*_0_ (*F*_3,660_ = 77.59, *p* < 0.0001), suggesting that *T*_body_ was intentionally regulated. Regulation of *T*_body_ above Δ*T*_0_ might also be occurring for BCHU and RIHU, but the number of high *T*_e_ measurements (*T*_e_ > Δ*T*_0_) for these species was limited (*n* = 15 and 7, respectively). No measurements of BLUH beyond Δ*T*_0_ were made, so their response to high *T*_e_ remains unknown.

## 4. Discussion

Our *T*_eye_ and behavioural data suggest that hummingbirds in this study must rely primarily on evaporative heat dissipation for body-temperature management when *T*_eye_ approaches *T*_e_ (hypothesis 1, figures 4 and 7). Because *T*_eye_ in the two smaller species was 3–4°C higher than the larger species, it is possible that the smaller species might be able to continue some passive heat dissipation at higher *T*_e_ than the larger species. We have also shown that *T*_e_ ≥ *T*_eye_ for up to greater than 6 h at both SC and EC (except for BCHU at EC), suggesting that heat dissipation during flight would require dependence on evaporative heat dissipation for extended periods during the day (hypothesis 2, figures 1 and 6). The mean hourly daytime *T*_e_ at HC and SWRS was generally 2–3°C lower so that some passive heat dissipation was possible on all but the hottest days. Finally, when BBLH (and possibly all our study species) in southeastern Arizona lose their ability to passively dissipate heat, they appear to actively regulate their *T*_body_ during hovering to avoid passive heat gain and protect themselves from hyperthermia (hypothesis 3, figure 7).

During summer, a thermal gradient for passive heat dissipation can be small or non-existent in some habitats for 6–10 h during the day (figure 6), requiring hummingbirds to rely heavily on evaporative heat dissipation and perhaps seek cooler microclimates within vegetation to cool themselves so that they are able to make short foraging flights to acquire the necessary energy to maintain their energy budgets. With climate change, predicted increases in yearly and daily *T*_e_ [35] could reduce the availability of protective microclimates making evaporative heat dissipation the only option for hummingbirds to control body temperature during foraging for much of the day [19].

While *T*_body_ is positively correlated with *T*_e_, *T*_HDA_ is controlled by the body core temperature and is relatively fixed across the range of *T*_e_ measured in this study (figure 2). Thus, as *T*_e_ increases Δ*T* and the effective size of the eye HDA decreases until passive heat dissipation is no longer possible (between 36 and 40°C for all species in this study). When *T*_e_ exceeds Δ*T*_0_, heat balance becomes challenging because of passive heat gain due to reversal of the thermal gradient, and the need to rely solely on evaporative heat dissipation for cooling. REWL accounts for 30–35% of total heat dissipation in all our study species at *T*_e_ ∼ 40°C [25], requiring the remaining 65–70% of generated metabolic heat to be dissipated via CEWL. While CEWL has never been measured in hovering hummingbirds, total evaporative water loss measured between 20 and 40°C on several hummingbird species at rest accounted for no more than 40% of the total heat dissipation [36,37], so CEWL would need to be substantially upregulated to balance the heat budget.

Substantial upregulation of CEWL at high *T*_e_ does occur in birds. At extremely high *T*_e_ (greater than 50°C) resting arid-zone columbids dramatically increase CEWL, allowing them to maintain body temperatures below their thermal endpoints [7]. However, evaporative heat dissipation at high *T*_e_ in smaller resting passerines is dominated by REWL (panting) [7,38]. Anecdotally, perching hummingbirds in the field on hot days are frequently seen panting, which could suggest an emphasis on REWL, but REWL alone is not sufficient to balance metabolic heat production during hovering. Hummingbirds have high water turnover rates (1.6–3.7× total body water [39–41]), much higher than predicted for their body mass [42], making them unique even among small birds that all have proportionally large skin surface relative to their mass; thus hummingbirds may be ideally adapted for upregulation of CEWL. A full understanding of the role EWL plays in heat balance will require laboratory measurements which are beyond the scope of this study.

Hummingbird species in this study showed differences in their response to high *T*_e,_ with the smaller species appearing to be more tolerant of high *T*_e_ than the larger species. Both BBLH and BCHU exhibited higher Δ*T*_0_, and slower decrease in Δ*T* and eye HDA length as *T*_e_ increased compared to BLUH and RIHU (figure 4). Higher temperature tolerance in the smaller species seems counterintuitive, and a physiological explanation is not clear. BBLH does exhibit the highest REWL of all the study species, but REWL in BCHU is similar to that in larger species [25]. BCHU is the only species for which eye HDA length shows no relationship to *T*_e_, but this is probably an artefact of the narrow *T*_e_ range over which measurements were made. Alternatively, the relatively high surface-to-volume ratio of BBLH and BCHU improves convective cooling due to induced air velocity from the wings [3]. Our results appear consistent with the pattern of relatively small hummingbird species specializing on desert environments (e.g. Costa's hummingbird (*Calypte costae*) or Lucifer's hummingbird (*Calothorax lucifer*)), so the relationship between body size and thermal tolerance merits broader comparative study.

In all species except BCHU, reduction in passive heat dissipation accelerated as *T*_HDA_ approached Δ*T*_0_ (figure 5). Because of the way we measured eye HDA length, the length would include some surface along the head outside the eye HDA proper [3]. As surface temperature outside an HDA is strongly influenced by *T*_e_ (figure 2), higher exposure to solar radiation could result in a higher *T*_body_, which would speed the decline of both Δ*T* and eye HDA length.

Variation in heat tolerance during hovering, at least in part, may be driven by behavioural differences among species. BLUH is highly territorial [43–45], and territorial defence behaviours (e.g. agonistic chases) could increase their exposure to solar radiation. RIHU is a trap-liner (travels between food sources in a regular, repeating pattern [30]) and was highly transient at our study sites, so frequent movements could also increase exposure to solar radiation. BCHU is non-territorial and acquires energy by competing with BBLH at HC and SC [46], RIHU at EC (D. Powers 2014, personal observation) and mostly by intruding on BLUH territories at SWRS [43–45], and their low fat storage and high frequency of torpor use [47] suggests that they might spend large amounts of time perching and only flying to feed or engage in courtship. Typical perching locations would protect them from exposure to solar radiation and slow down the decrease in Δ*T* and eye HDA length. While BBLH is considered a trap-liner [20], their high degree of site fidelity based on banding sessions at our study sites (D. Powers 2013, personal observation) suggests they are less transient than RIHU, and might benefit from extended periods of perching in a manner similar to that of BCHU.

*T*_e_ can be sufficiently high to reach or exceed Δ*T*_0_ from 5 to 9 h during the day for all species except BCHU (figure 6). Sonoita Creek (Patagonia) had the highest daytime *T*_e_ and exhibited the longest periods where Δ*T*_0_ was exceeded, resulting in lengthy periods of reduced activity in BBLH (S. Wethington 2013, unpublished point count data). In the Chiricahua sites, *T*_e_ was cooler, but still could exceed BLUH and RIHU Δ*T*_0_ for several hours. RIHU were transient at our sites, so it was difficult to determine how long individuals remained at our feeders. Individual RIHU tended to perch in nearby trees, making foraging bouts short, which could reduce total heat production during flight, thereby allowing intermittent foraging even at higher *T*_e_. No BLUH measurements were recorded in this study between 12.00 and 17.30 at SWRS, suggesting a substantial reduction of flight activity during the warmest times of day. Interestingly, *T*_e_ values during this period of BLUH inactivity roughly corresponds to the range of *T*_e_ values over which most of our BCHU measurements occurred. It is possible that the BCHU ability to maintain a favourable Δ*T* over the range of *T*_e_ we measured allowed them to forage frequently during midday with minimal disturbance from BLUH, and could be an example of temporal/thermal niche partitioning. Temporal partitioning has been suggested as a way for a wide range of species who use the same resource to coexist [48–50]. Thermal niche partitioning has been proposed as a mechanism allowing coexistence of similar ectotherms, but a recent review of these studies suggests that they lack sufficient experimental rigor to conclusively demonstrate the existence of this type of niche sharing [51]. While our data for BLUH and BCHU are insufficient to claim evidence for either form of niche partitioning, this theme would be a useful direction for future study in the hope of better understanding rules of assembly in hummingbird communities [52].

We did not directly measure hummingbird behaviour, but our measurements of *T*_body_ suggest behavioural shifts at high *T*_e_ (figure 7). Below Δ*T*_0_, *T*_body_ increases linearly similar to that observed in our controlled, captive-bird studies (figure 2). In BBLH, as *T*_e_ approaches and exceeds Δ*T*_0_, *T*_body_ becomes constant at approximately 39°C, slightly below both their Δ*T*_0_ and likely hummingbird body temperature [18,53]. The lack of correlation between *T*_body_ and *T*_e_ when Δ*T*_0_ is reached is probably due to behaviour regulation of *T*_body_ to minimize passive heat gain. BBLH at our sites congregated near riparian areas which could provide numerous thermal refugia near feeding stations allowing birds to minimize exposure to solar radiation and perch to dissipate heat accumulated during foraging flights. All *T*_body_ measurements for BLUH were well below Δ*T*_0_, and are consistent with other measurements in suggesting lower thermal tolerance than the other species in this study.

By taking advantage of the thermal variability found in the various microclimates of an ecosystem, birds could mitigate the impact of warming temperatures associated with climate change [19]. Over the past several years, use of thermal microclimates as protection from high environmental temperature has been studied extensively in ectotherms [54], so it stands to reason that this behavioural strategy would also be important for endotherms. The few measurements we obtained for BCHU and RIHU at or above Δ*T*_0_ suggest a pattern of behavioural regulation for *B*_body_ similar to what we observed in BBLH, and is commonly observed in ectotherms.

## 5. Conclusion

Populations of hummingbird species in this study are common and the primary breeding species at our study sites, yet they are seemingly obligated to make physiological and/or behaviour adjustments at high temperature as a thermoregulatory strategy to mitigate the effects of high body-heat load. The two smaller species in this study appear to tolerate higher temperatures better than the larger species as indicated by their higher Δ*T*_0_, and in the case of BCHU, the ability to maintain a favourable Δ*T* across the full range of measured *T*_e_. Reasons for differences in temperature tolerance between the smaller and larger species are not clear. One explanation is that the larger species do not require high temperature tolerance. Alternatively, smaller species might be taking advantage of their relatively higher surface-to-volume ratio to improve self-induced convective cooling from forced air movement from the wings. Regardless, a full understanding of (i) how hovering hummingbirds manage body heat at high temperature and (ii) the upper limits of temperature tolerance will require measurements of evaporative heat dissipation during hovering, and specifically measurements of CEWL.

## Supplementary Material

MCMCglmm Model Parameters

## Data accessibility

All data are available at Dryad: http://dx.doi.org/10.5061/dryad.c0765 [55].
